# Mapping sentence comprehension and syntactic complexity: evidence from 131 stroke survivors

**DOI:** 10.1093/braincomms/fcae379

**Published:** 2024-11-15

**Authors:** Nicoletta Biondo, Maria V Ivanova, Alexis L Pracar, Juliana Baldo, Nina F Dronkers

**Affiliations:** Department of Psychology, University of California, Berkeley, Berkeley, CA 94720, USA; Basque Center on Cognition, Brain, and Language, Donostia 20009, Spain; Department of Psychology, University of California, Berkeley, Berkeley, CA 94720, USA; Department of Psychology, University of California, Berkeley, Berkeley, CA 94720, USA; Veteran Affairs Northern California Health Care System, Martinez, CA 94553, USA; Department of Psychology, University of California, Berkeley, Berkeley, CA 94720, USA; Department of Neurology, University of California, Davis, Sacramento, CA 95817, USA

**Keywords:** comprehension, aphasia, lesion, disconnection, syntax

## Abstract

Understanding and interpreting how words are organized in a sentence to convey distinct meanings is a cornerstone of human communication. The neural underpinnings of this ability, known as syntactic comprehension, are far from agreed upon in current neurocognitive models of language comprehension. Traditionally, left frontal regions (e.g. left posterior inferior frontal gyrus) were considered critical, while more recently, left temporal regions (most prominently, left posterior middle temporal gyrus) have been identified as more indispensable to syntactic comprehension. Syntactic processing has been investigated by using different types of non-canonical sentences i.e. those that do not follow prototypical word order and are considered more syntactically complex. However, non-canonical sentences can be complex for different linguistic reasons, and thus, their comprehension might rely on different neural underpinnings. In this cross-sectional study, we explored the neural correlates of syntactic comprehension by investigating the roles of left hemisphere brain regions and white matter pathways in processing sentences with different levels of syntactic complexity. Participants were assessed at a single point in time using structural MRI and behavioural tests. Employing lesion–symptom mapping and indirect structural disconnection mapping in a cohort of 131 left hemisphere stroke survivors, our analysis revealed the following left temporal regions and underlying white matter pathways as crucial for general sentence comprehension: the left mid-posterior superior temporal gyrus, middle temporal gyrus and superior temporal sulcus and the inferior longitudinal fasciculus, the inferior fronto-occipital fasciculus, the middle longitudinal fasciculus, the uncinate fasciculus and the tracts crossing the most posterior part of the corpus callosum. We further found significant involvement of different white matter tracts connecting the left temporal and frontal lobes for different sentence types. Spared connections between the left temporal and frontal regions were critical for the comprehension of non-canonical sentences requiring long-distance retrieval (spared superior longitudinal fasciculus for both subject and object extraction and spared arcuate fasciculus for object extraction) but not for comprehension of non-canonical passive sentences and canonical declarative sentences. Our results challenge traditional language models that emphasize the primary role of the left frontal regions, such as Broca’s area, in basic sentence structure comprehension. Our findings suggest a gradient of syntactic complexity, rather than a clear-cut dichotomy between canonical and non-canonical sentence structures. Our findings contribute to a more nuanced understanding of the neural architecture of language comprehension and highlight potential directions for future research.

## Teaser

Biondo *et al*. investigated sentence comprehension in 131 left hemisphere stroke survivors. They found that the left temporal regions and distinct temporo-frontal white matter pathways are necessary for the comprehension of different sentence structures. Results suggest a gradient of syntactic complexity, challenging traditional views relying on simplistic canonical versus non-canonical distinctions.

## Graphical Abstract

**Graphical Abstract:**
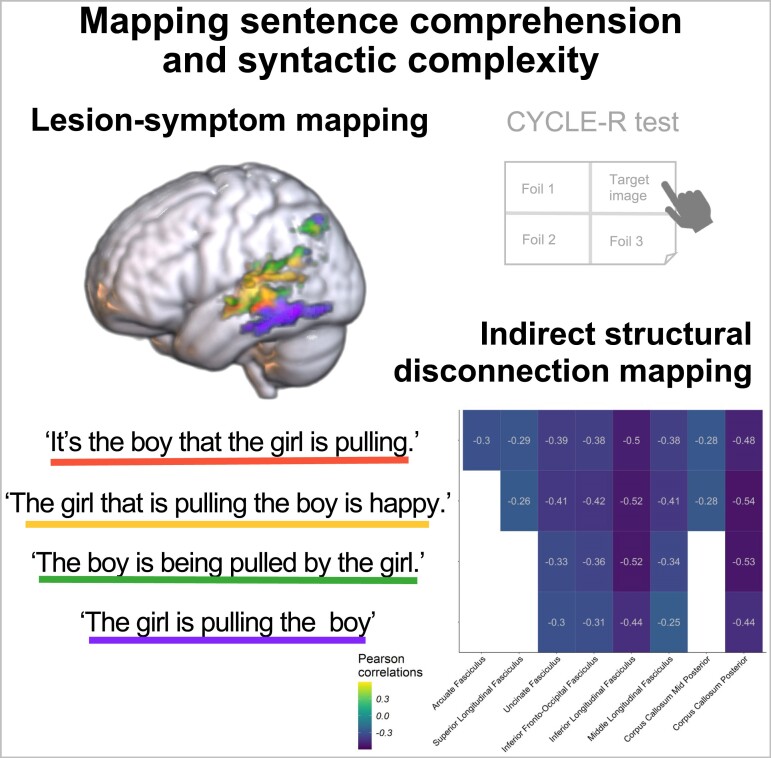

## Introduction

Syntactic comprehension is a unique feature of human communication allowing us to understand and interpret the grammatical structures and rules that define how words are organized in a sentence to convey a specific meaning. Depending on the structure of the sentence, this process can be straightforward or very difficult. For example, simple, canonical sentences such as ‘The girl kisses the boy’ are easy to comprehend because we can simply rely on the order and meaning of the content words (e.g. girl kisses boy). In contrast, non-canonical sentences such as ‘It’s the boy that the girl kisses’ might convey a similar meaning but are more difficult to comprehend because we have to recognize that the boy is the one being kissed, not doing the kissing. In other words, we cannot rely solely on word order to comprehend the meaning of the sentence.

In this paper, we set out to identify grey and white matter structures that are most critical for the auditory comprehension of sentences with varying syntactic complexity. We used both lesion–symptom mapping (LSM) and indirect structural disconnection mapping in a large cohort of left hemisphere stroke survivors with a range of comprehension deficits. LSM allows us to link behavioural performance on specific tasks to the presence/absence of a lesion in specific parts of the brain, providing a statistical map of areas critical for the behaviour under examination.^1^ Indirect structural disconnection mapping is an approach used to study the impact of brain lesions on the connectivity of white matter pathways. This method provides estimates of structural disconnection in the absence of diffusion-weighted imaging data by integrating spatial lesion data obtained from brain-damaged individuals with the normative white matter connectome data obtained from healthy individuals.^2^

The goal of our study was 2-fold. First, we aimed to identify the left hemisphere brain regions and fibre pathways that are most critical for general sentence comprehension. Second, we wanted to explore whether sentences with varying levels of syntactic complexity, beyond the coarse differentiation of ‘canonical versus non-canonical’, rely on similar or different brain regions and white matter pathways.

Current neurocognitive models of language processing^3-6^ that incorporate findings from multiple sources (e.g. functional MRI, magnetoencephalography, patient data) outline an extensive network of different peri-Sylvian brain regions involved in language comprehension. However, the neural underpinnings of specific processes like the ones involved in syntactic comprehension are still hotly debated. For all these models, left temporal regions are involved in syntactic comprehension.^3-6^ The left posterior superior temporal gyrus (STG) is believed to be involved in early stages of syntactic processing, namely in the integration of phonological and syntactic information, such as in the mapping of sounds to words^4-6^ and in initial structure building.^4,6,7^ The more anterior part of the STG is also mentioned as relevant for initial local sentence structure building.^3^ The left posterior middle temporal gyrus (MTG) is thought to be devoted to building sentence structure and to the integration of semantic and syntactic information,^3^ such as in the retrieval of semantic information from memory and the mapping of this information into syntactic structures to comprehend the general meaning of the sentence.^4-6^ All models also suggest that the left posterior MTG plays an important role in the processing of complex sentence structures, such as passives and relative clauses.^3-6^ Finally, the left posterior superior temporal sulcus (STS) is thought to be involved in complex syntactic processing, such as the resolution of syntactic ambiguities,^6^ the processing of discourse-related information necessary for referential dependencies such as the processing of pronouns^4^ and the integration of sentence-level prosody with syntactic structure and meaning.^3,5^

At the same time, there is less consensus on the role of left frontal regions, namely the posterior inferior frontal gyrus (pIFG) or Broca’s area (most commonly defined as BA 44 and 45), for syntactic comprehension. For some models, Broca’s area is crucial for core syntactic operations such as ‘merge’^7,8^ or syntactic unification,^4^ the basic process of combining two words together to create a meaningful syntactic unit (e.g. ‘the’ and ‘girl’). Other accounts further specify that Broca’s area is critical for processing complex sentences where words do not follow the canonical word order^3,9^ and perhaps require additional working memory resources.^10-15^ By other models, the left pIFG does not play a critical role during syntactic comprehension but rather performs general top-down cognitive supervisory functions that are non-syntax specific.^5,16^ Still other models suggest it is not critical at all for syntactic comprehension and only relevant for sentence production.^6^

LSM analyses help clarify the crucial role of specific portions of the brain for the successful performance of specific language functions. Although previous LSM studies consistently support the role of left temporal regions in syntactic comprehension,^11,17-28^ they provide a more heterogeneous picture on the role of left frontal regions. Among LSM studies that do report the involvement of left frontal regions in sentence comprehension,^11,18,20,24^ different regions within the frontal lobe have been identified as critical, including more pIFGs, namely pars opercularis and triangularis,^20^ pars triangularis exclusively,^24^ the frontal operculum^18^ or more anterior portions of the inferior frontal gyrus (IFG), namely BA46 and BA47.^11^ It should be noted that these studies differed methodologically in the type of patients tested (acute^18,20^ versus chronic^11,24^ stroke), in the type of task adopted (sentence–picture matching,^11,20^ yes/no comprehension questions,^18^ token test^24^), in the type of covariates included in the analyses to control for individual differences (no covariates,^11,20^ age,^18^ lesion volume^24^) and in the LSM algorithms used.

Syntactic comprehension has traditionally been assessed by comparing the comprehension of ‘simple’ canonical sentences with the comprehension of ‘complex’ non-canonical sentences, such as relative clauses and passive constructions. Canonical sentences typically follow the word order of the language, such as subject–verb–object in English active sentences (e.g. ‘The girl kisses the boy’). Among the non-canonical sentences, which do not follow the prototypical word order of the language, the most studied ones contain passives or relative clauses. Passive clauses reorganize the syntactic structure of a sentence to emphasize the recipient or ‘patient’ of an action rather than the doer or ‘agent’, which can be silent (not present in the sentence) or introduced by the preposition ‘by’ (e.g. ‘The boy was kissed by the girl’), in English. Relative clauses are subordinate clauses that modify and provide additional details about a noun (the antecedent) within a main clause (e.g. ‘The boy that the girl kisses __ is tall’). They typically begin with a relative pronoun (e.g. who, which, that) and contain a gap (or trace) that corresponds to the position of the antecedent in the relative clause. The gap can be in subject position (e.g. ‘The boy that __ kisses the girl is tall’) or in object position (e.g. ‘The boy that the girl kisses __) thus leading to subject-extracted and object-extracted relative clauses. Both passive constructions and relative clauses differ from canonical sentences since they both require a reshuffling of the elements within the sentence. However, the comprehension of these two types of non-canonical sentences involves different language mechanisms, which may rely on different neural underpinnings. In order to successfully comprehend passive sentences, the ability of assigning thematic roles,^29,30^ without following the prototypical subject-agent object-patient role assignment, needs to be spared. In order to successfully comprehend relative clauses, both the ability of assigning thematic roles and the ability of retrieving the right antecedent^9,31^ that was processed and stored in memory need to be spared.

Previous LSM studies investigated syntactic comprehension in different ways. Some studies investigated the comprehension of different canonical and non-canonical sentences separately,^11,17^ while others grouped together different types of canonical sentences, such as declaratives and subject relative clauses and different types of non-canonical sentences such as passives and object relative clauses.^19,20,22,25,27^ Other studies focused exclusively on the comprehension of canonical declarative sentences^24,28^ or on the comprehension of non-canonical sentences.^23,25-27^ Finally, other studies considered canonical and non-canonical sentences altogether.^18,21^ It is therefore difficult to draw clear conclusions on the role of specific regions for the comprehension of different sentence types that may rely on different language mechanisms.

Successful comprehension not only relies on cortical regions but is also likely dependent on white matter tracts.^11,32-35^ These include the arcuate fasciculus (AF) and the superior longitudinal fasciculus (SLF) that connect the frontal cortex with temporal and inferior parietal regions, respectively. The inferior longitudinal fasciculus (ILF) and the inferior frontal-occipital fasciculus (IFOF) pass through the temporal lobe. The former connects the occipital lobe with the anterior portions of the temporal lobe. The latter connects the middle and inferior frontal gyri and the inferior orbitofrontal cortex. Other relevant tracts are the uncinate fasciculus (UF), which connects the anterior portion of the temporal lobe with the inferior frontal cortex, and the middle longitudinal fasciculus (MdLF), which connects the inferior parietal lobe with the anterior STG and STS. Current models of sentence comprehension consider the left AF and SLF to be critical for syntax processing and for computing hierarchical dependencies among words.^3-5,7^ Temporal lobe tracts are generally associated with the processing of meaning/semantic information, although the exact functional role of each tract is still unclear. The extreme capsule is often cited for the processing of semantic information,^3,4^ while the UF is associated with the processing of simple rule-based sequences such as local syntactic structure building.^3,7^ A recent meta-analysis^36^ tested the role of several white matter tracts in different linguistic domains by analysing diffusion metrics of 46 studies (*n* = 1353 aphasics). The meta-analysis showed the involvement of the left ILF and IFOF for general sentence-level comprehension, while the SLF and the AF were involved in syntactic processing. It should be noted however that the analysis of syntactic processing included a wide variety of language tests varying greatly in type of tasks (that included both production and comprehension) and involving a wide variety of sentence types. Therefore, we cannot exclude that the comprehension of different complex sentences relies on the integrity of different tracts. Finally, white matter connections to homologous right hemisphere regions might also assist with language comprehension.^37-42^ In particular, the posterior part of the corpus callosum (CC), the isthmus and the splenium, which contains fibres that connect the posterior temporal lobes,^43,44^ has been implicated in sentence comprehension.^32,45-47^

In this study, we aimed to provide a more fine-grained investigation of syntactic comprehension. We addressed the limitations of the existing literature in four different ways. First, we tested syntactic comprehension in a large cohort of post-stroke individuals (*n* = 131), which provided sufficient statistical power to investigate the role of many different left hemisphere cortical areas. Second, we performed both univariate and multivariate LSM analyses and included covariates to control for individual differences.^48-53^ Third, we computed the severity of tract-level disconnection^54^ in the major tracts thought to be involved in syntactic comprehension and correlated those measures with behaviour, in order to assess the role of different white matter pathways for the comprehension of different sentence structures. Finally, we analysed the comprehension of different types of canonical and non-canonical sentences separately. This allowed us to explore potential differences in the neural underpinnings of sentences with varying levels of syntactic complexity, rather than relying simply on the canonical/non-canonical distinction.

## Materials and methods

### Participants and measures

Participants included 131 individuals (32 females and 99 males) who sustained a single left hemisphere stroke at least 6 months before being tested and scanned. Our sample size exceeds the minimum estimate recommended for reliable LSM results.^51^ All participants were right handed (based on Edinburgh Handedness Inventory), had native-like proficiency in English prior to their stroke, had more than 8 years of education and normal or corrected-to-normal hearing and vision. Participants with additional neurologic diagnoses (e.g. multiple strokes, Parkinson’s, dementia) and significant psychiatric disorders (e.g. schizophrenia, bipolar disorder) were excluded. Data from 64 of the participants were included in a prior study on sentence comprehension.^11^ A summary of the participants’ demographic data is provided in Table 1. All participants provided their informed consent, and research was approved by the Institutional Review Boards at the VA Northern California Health Care System and the University of California, Berkeley in line with the Helsinki Declaration.

**Table 1:** Participants’ details

Variables	Range, mean, and standard deviation
Age	Range 31–86 years; *M* = 62.7; SD = 11.7
Education	Range 10–22 years; *M* = 15.1; SD = 2.6
Time post-onset	Range 7–329 months; *M* = 59; SD = 67.5
Lesion volume	Range 15–56 870 mm^3^; *M* = 12 898; SD = 11 453
Aphasia Quotient (AQ) of the Western Aphasia Battery (WAB)	Range 10–100 score; *M* = 74.8; SD = 26.6
WAB subtests	Information content: range 0–10 score; *M* = 7.7; SD = 2.9 Fluency: range 0–10 score; *M* = 7.4; SD = 2.9 Yes/no questions: range 12–60 score; *M* = 55.2; SD = 8.1 Auditory word recognition: range 9–60 score; *M* = 52.4; SD = 12.4 Sequential commands: range 2–80 score; *M* = 60.4; SD = 22 Repetition: range 0–100 score; *M* = 69.9; SD = 34.5 Object naming: range 0–60 score; *M* = 45.1; SD = 20.3 Word fluency: range 0–20 score; *M* = 9; SD = 6.3 Sentence completion: range 0–10 score; *M* = 7.5; SD = 3.6 Responsive speech: range 0–10 score; *M* = 7.3; SD = 3.9
CYCLE-R comprehension accuracy	Simple: range 20–100 score; *M* = 90.1; SD = 16.7 Passive: range 10–100 score; *M* = 77.3; SD = 26.7 Subject extraction: range 13–100 score; *M* = 71.4; SD = 27.8 Object extraction: range 0–100 score; *M* = 64.9; SD = 28.7

We report the range (min–max), the mean and the standard deviation values for each variable.

### Behavioural data

Participants’ language performance was evaluated with the Curtiss–Yamada Comprehensive Language Evaluation (CYCLE-R^55^) test. On the CYCLE-R, participants are asked to listen to a sentence presented verbally by the experimenter and select the picture that best matches the meaning of the sentence they just heard from an array of either three or four line drawings. The CYCLE-R includes a series of subtests, and each subtest includes five sentences that target a particular syntactic structure.

The CYCLE-R subtests analysed in the current study included a wide range of declarative sentences with varying levels of syntactic complexity (see Table 2). First, we considered all sentence types together to assess the neural underpinnings of general sentence-level comprehension. Then, we divided the subtests into four main groups. The ‘simple’ group contained sentences following the canonical word order, such as subject–verb–object in English active sentences. The ‘passive’ group contained sentences where the element undergoing the action (generally in object position) becomes the subject of the sentence (e.g. ‘The boy is being pushed by the girl’). The ‘object extraction’ group contained sentences where the displaced element is the object of a relative clause (e.g. ‘It’s the clown that the girl chases’). The ‘subject extraction’ group contained sentences where the displaced element is the subject of a relative clause (e.g. ‘The girl who is pushing the boy is happy’).

**Table 2:** List of CYCLE-R subtests used to assess participants’ sentence comprehension

Sentence groups	CYCLE-R subtests	Example	Sample picture contrast			
			Target	Foil 1	Foil 2	Foil 3
Simple	Possession	‘The clown has a balloon’	Clown with balloon	Girl with balloon	Clown with flower	(No third foil)
	Simple declaratives	‘The boy is jumping’	Boy jumping	Girl jumping	Boy standing	(No third foil)
	Active voice order	‘The girl is pulling the boy’	Boy sitting in wagon, girl pulling wagon	Girl sitting in wagon, boy pulling wagon	Boy sitting in wagon, dog pulling wagon	Girl and boy pulling wagon together
Passive	Passive voice Order I	‘The girl is being kicked’	Boy kicking girl (ball in background)	Girl kicking boy (ball in background)	Girl kicking ball	(No third foil)
	Passive voice Order II	‘The girl is being kicked by the boy’	Boy kicking girl (ball in background)	Girl kicking boy (ball in background)	Clown kicking boy (ball in background)	Boy kicking clown (ball in background)
Subject extraction	Subject relatives ending in N–V	‘The girl who is pushing the boy is happy’	Happy girl pushing angry boy sit on swing	Angry girl pushing happy boy sit on swing	Angry boy pushing happy girl sit on swing	Happy boy pushing angry girl sit on swing
	Object (O–S) relative clauses	‘The girl is chasing the clown who is big’	Small girl chasing big clown	Big girl chasing small clown	Big clown chasing small girl	Small clown chasing big girl
	Double embedding I	‘The clown that is big has a balloon that is blue’	Big clown holding blue balloon	Small clown holding blue balloon	Big clown holding red balloon	Small clown holding red balloon
Object extraction	Object clefting	‘It’s the boy that the girl kicks’	Girl kicking boy	Boy kicking girl	Girl kicking clown	Boy kicking clown
	Object relatives (O-O) with relativized object	‘The girl is kissing the boy that the clown is hugging’	Girl kissing boy; clown hugging boy (girl standing; boy and clown sitting on bench)	Girl kissing boy; boy hugging clown (girl standing; boy and clown sitting on bench)	Girl kissing clown; boy hugging clown (girl standing; boy and clown sitting on bench)	Boy kissing girl; clown hugging girl (boy standing; girl and clown sitting on bench)

### Neuroimaging data

#### Lesion mapping

**Figure 1:**
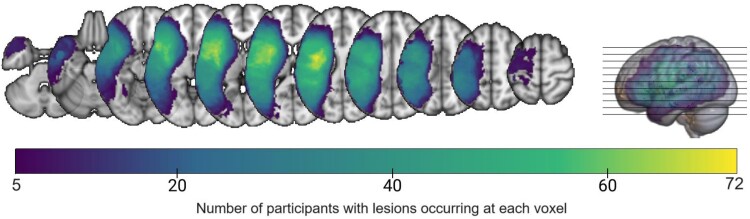
**Lesion overlay.** Lesion overlay map of the voxels that entered the LSM analyses.

Participants’ lesions were reconstructed manually from MRI or CT data acquired at least 6 months post-stroke. Data acquired before 2012 (*n* = 96) were previously reported in the study by Baldo *et al*.^56^ High-resolution T1-weighted structural 3D MRI scans were acquired for 43 participants using a 1.5 T Phillips Eclipse scanner. The T1-weighted images were collected with a Spoiled Gradient Recall sequence encompassing 212 coronal slices. Lesions, whose extents were confirmed using T2-weighted and fluid-attenuated inversion recovery (FLAIR) images, were manually delineated directly on the T1 digital MRI images utilizing MRIcro software.^57^ These lesion reconstructions were then aligned with the montreal neurological institute (MNI) template using the standard non-linear spatial normalization procedure from SPM2 incorporating a cost function masking procedure to mitigate distortions due to the presence of lesions.^58^ In cases where 3D digital MRI images were unavailable, lesions were traced from hard-copy CT (*n* = 31) or MRI films (*n* = 22). The CT images were acquired using a Siemens Somatom Emotion 16 CT scanner with 3 × 3 × 3 mm resolution, while the MRI images were similarly obtained using the 1.5 T scanner. Lesions were drawn manually onto an atlas-based^59^ 11-slice standardized template by a board-certified neurologist who was blinded to the patient’s behavioural data and study hypotheses (for reliability of these methods, see studies by Friedrich *et al*.^60^ and Knight *et al*.,^61^). These templates were digitized and transformed into MNI space using SPM5. This transformation involved aligning slices from both templates with 50 control point pairs to match anatomical features, followed by a local weighted mean transformation using the *cpselect*, *cp2tform* and *imtransform* functions in Matlab 6.5. These functions were used to warp all lesion reconstructions from the 11-slice template into MNI space. Data acquired after 2012 (*n* = 35) were collected either on a 3 T Siemens Verio or Trio scanner using an magnetization-prepared rapid gradient echo (MP-RAGE) sequence with 1 × 1 × 1 mm voxel size. The participants’ lesions were traced directly onto the patient’s native T1-weighted images manually using the MRIcron software^62^ or ITK-SNAP.^63^ The T2-weighted and FLAIR images (when available) were co-registered with the T1 images to verify the extent of the lesions. T1 images and binary lesion masks were normalized to an MNI template using a modified version of the unified segmentation/normalization algorithm implemented in SPM8 with cost function masking of the lesion (*Seg* toolbox in the SPM8 distribution^64^). This algorithm was customized to optimize the normalization of deep white matter and ventricles by using an age-relevant template and, additionally, by incorporating a head model.^13,51,65^ This choice provided a tighter fit to the template space without distorting overall brain anatomy.^64^ The lesion masks were converted to standard MNI space with a 2-mm isovoxel resolution. The overlay of patients’ lesions used in the LSM analyses is presented in Fig. 1 and shows that a large portion of the middle cerebral artery distribution was included.

#### Indirect structural disconnection mapping

The Lesion Quantification Toolkit^54^ was used to model the effects of each lesion on the typical white matter connectome to estimate the degree of white matter tract disconnection. The Toolkit is based on the HCP-842 atlas, which was built by using high-resolution diffusion MRI data collected from 842 healthy participants.^66^ Each participant’s lesion and the atlas’ tracts were first embedded in the same space to identify the subset of streamlines that pass through the lesion-occupied volume. The percentage of disconnection severity was then determined by calculating the proportion of disconnected streamlines relative to the total number of streamlines, separately for each tract.

### Statistical analysis

#### Behavioural

Patients’ auditory comprehension accuracy on the CYCLE-R was analysed with logit mixed-effect models,^67^ with Sentence Group as a fixed effect, and random intercepts for the subject grouping factor.^68^ Potential inter-relationships among the comprehension of different sentence types were assessed using Pearson correlations. Two of 131 participants did not have accuracy scores for the subject extraction and object extraction sentence groups. Pairwise deletion was chosen to preserve as much information as possible for each analysis. List-wise deletion of those missing values did not significantly affect the results.

#### Lesion–symptom mapping

Both univariate and multivariate LSM analyses were conducted to relate auditory comprehension performance on the CYCLE-R to underlying lesion data, using methods described in detail in the study by Ivanova *et al*.^51^ Univariate LSM^1^ provides a statistical comparison of behaviour across patients with and without a lesion on a voxel-by-voxel basis. Only voxels that were lesioned in at least five participants were included in the analysis. Lesion masks had a 4-mm smoothing. In order to account for false positive results due to the large number of voxels being sampled, permutation testing was applied.^48,49,69^ In particular, we used a continuous permutation-based family-wise error rate (FWER) correction set to the 125th largest *t*-value with 1000 permutations (T-nu = 125; *n* = 125 corresponds to 1 cm^3^ when working with 2-mm-sided voxels), with a final threshold set at *P* = 0.05.^49,51^ The same data were also analysed with multivariate LSM, consistent with current recommendations in the literature.^51^ Multivariate LSM considers the effects of all lesioned voxels jointly in a unified model. For the multivariate analysis, we used the support vector regression–based (SVR) method with standard hyper-parameter values commonly used in the field.^70,71^

**Figure 2:**
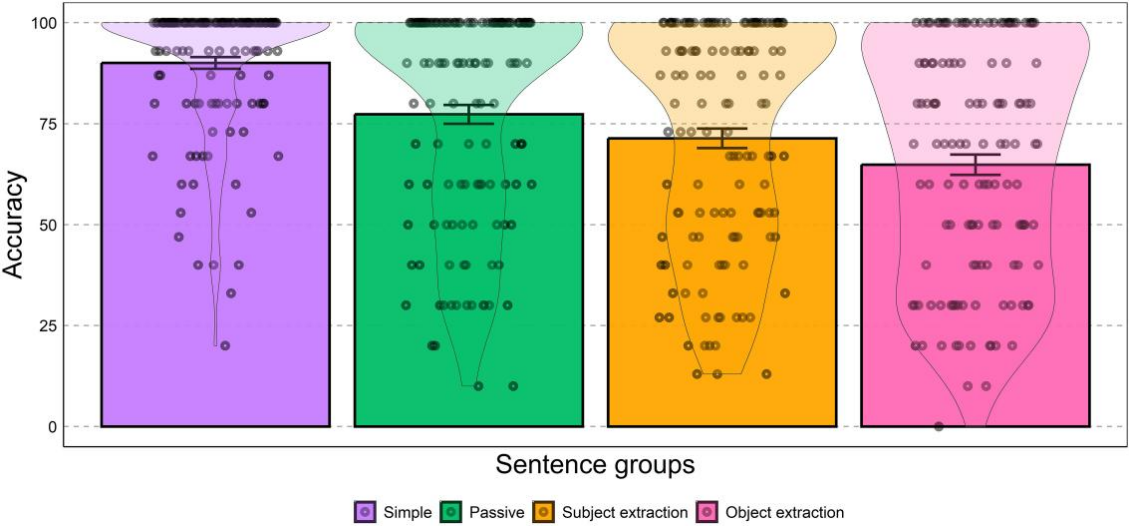
**Behavioural data.** Mean accuracy data (histogram), SEs (bars) and data distribution (violin plots) for each sentence group. Each data point represents the behaviour of each participant in our sample.

In all LSM analyses, we included the following demographic variables as covariates: age, years of education, months post-stroke and lesion size.^51,52^ Analyses that did not include lesion size as a covariate are reported in Supplementary Table 2 and Fig. 2. In order to address the concern of our results being influenced by the task and/or representing lexical-semantic rather than syntactic deficits, we ran an extra analysis. In this analysis, we removed the CYCLE subtest targeting lexical-semantic processing (‘Possession: The clown has a balloon’) from the ‘Simple’ sentence group and added it to the list of covariates.

All LSM analyses were implemented with the freely available CLSM Matlab package.^72^

#### Tract disconnection analyses

To evaluate the impact of tract-level disconnection severity on auditory sentence comprehension, we performed Pearson correlations analyses, Bonferroni corrected for the number of tracts (alpha = 0.0038). The tracts included the AF, the SLF, the UF, the IFOF, the ILF and the MdLF. We also analysed the five subsections of the CC (anterior: CCAneror, mid-anterior: CCMdAneror, central: CCCenra, mid-posterior: CCMdPoeror and posterior: CCPoeor) to investigate the role of inter-hemispheric connections. Finally, the frontal aslant tract (FAT) and the corticospinal tract (CST) were analysed as control tracts that were not expected to play a role during syntactic comprehension.

## Results

### Behavioural

The mean, standard error (SE) and distribution of the accuracy data for each sentence group is reported in Fig. 2. Comprehension of simple sentences was more accurate than the comprehension of passives (estimate: 2.36, SE: 0.52, z = 4.56, *P* < 0.0001), subject extraction (estimate: 2.66, SE: 0.53, *Z* = 5.04, *P* < 0.0001) and object extraction (estimate: 3.61, SE: 0.56, *Z* = 6.51, *P* < 0.0001). Comprehension accuracy of different types of complex structures also differed: sentences containing object extraction were more difficult to comprehend (resulting in lower scores) compared with sentences containing subject extraction (estimate: −0.96, SE: 0.36, *Z* = −2.66, *P* < 0.01) and passives (estimate: −1.25, SE: 0.37, *Z* = −3.34, *P* < 0.001). Comprehension of subject extraction and passives did not differ statistically (estimate: −0.29, SE: 0.36, *Z* = −0.11, *P* = 0.42).

The Pearson correlation analyses exploring the relationship between different sentence groups are reported in Table 3. Accuracy in comprehension of simple sentences was correlated with comprehension of sentences containing passives, subject or object extraction. In other words, participants with lower accuracy in the comprehension of simple sentences also had lower accuracy in the comprehension of non-canonical sentences. Similarly, participants with lower accuracy scores in the comprehension of passives also had lower scores in the comprehension of sentences containing subject extraction and/or object extraction.

**Table 3:** Pearson correlations between comprehension accuracy for different sentence groups

	Simple	Passive	Subject extraction	Object extraction
Simple	1	0.77	0.69	0.67
Passive		1	0.86	0.78
Subject extraction			1	0.84
Object extraction				1

### Lesion–symptom mapping

**Figure 3:**
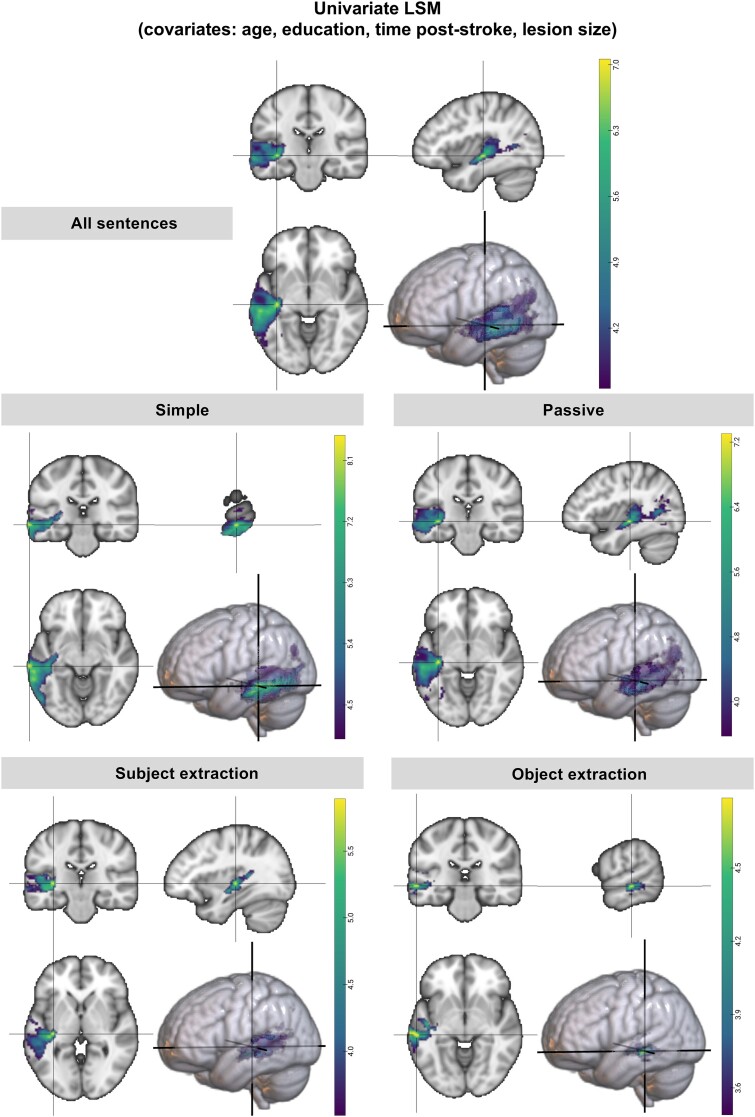
**LSM analysis (covariates: age, time post-stroke, education, lesion volume).** Univariate LSM maps for the comprehension of different groups of sentences from the CYCLE-R test, with age, months post-onset, education and lesion volume as covariates. The coloured bar represents the range of significant *t*-values following the continuous permutation-based FWER correction for multiple comparisons.

The results of the univariate LSM analyses for all sentences considered together and each sentence group separately (simple, passive, subject extraction, object extraction) are summarized in Fig. 3. For each LSM analysis, we report the maximum statistic location (Max_LSM_^51^), namely the peak coordinates [px, py, pz] of the significant cluster identified by each analysis within the text. Parcels from the Brainnetome Atlas^73^ that were covered by the results of each LSM analysis are reported in the Supplementary Table 1.

The results of the univariate LSM analysis assessing crucial neural underpinnings for general sentence-level comprehension identified portions of the left mid-posterior temporal lobe and left inferior parietal lobe, namely the mid-posterior STS and STG, the MTG, the inferior temporal gyrus (ITG) and the inferior parietal lobule (IPL), as well as underlying white matter (Max_LSM_ [−38, −22, −6] IFOF/ILF). Simple sentence comprehension was associated with more inferior portions of the temporal lobe, namely the temporo-occipital region of the MTG and the posterior and temporo-occipital division of the ITG (Max_LSM_ [−70, −22, −16] MTG, caudal area 21). Conversely, the processing of more complex sentences involved the mid-posterior left MTG and more superior portions of the temporal lobe such as the left STG, STS and underlying white matter (Max_LSM_ passives: [−38, −24, −6] IFOF/ILF; subject extraction: [−34, −24, 0] IFOF and object extraction: [−60, −28, −6] anterior STS). A portion of the IPL, the angular gyrus, was also found to be relevant for the comprehension of passive sentences. The multivariate LSM analyses identified significant LSM clusters in four of the five sentence group analyses (see Supplementary Fig. 1 and Table 1) with similar foci to the univariate analyses, providing additional confirmation of the findings.^51^

**Figure 4:**
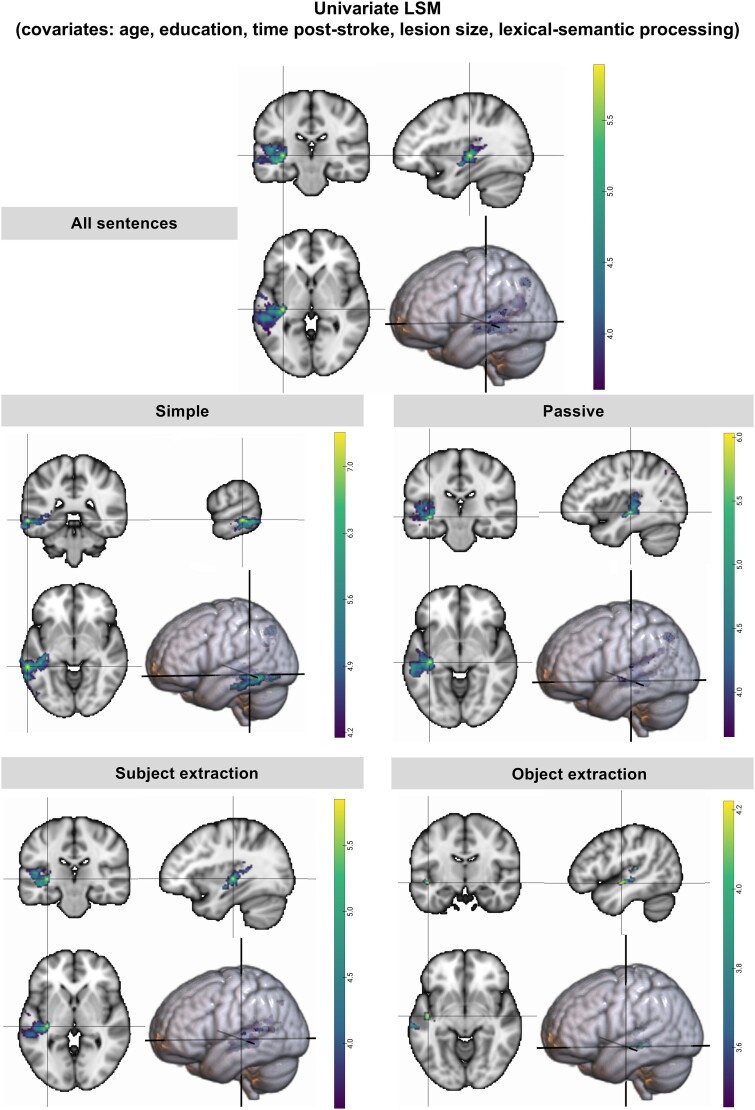
**Additional LSM analysis with lexical-semantic processing as covariate.** Univariate LSM maps for the comprehension of different groups of sentences from the CYCLE-R test, with age, months post-onset, education, lesion volume and lexical-semantic processing as covariates. The coloured bar represents the range of significant *t*-values following the continuous permutation-based FWER correction for multiple comparisons.

The results of the extra LSM analyses controlling for lexical-semantic processing abilities showed smaller clusters but with similar foci, as shown in Fig. 4 (a full list of Brainnetome parcels was provided in the Supplementary Table 3). The results of the univariate LSM analysis considering all sentences identified portions of the left temporal lobe and inferior parietal lobe, namely the anterior and mid-posterior STS and STG, the MTG, portions of the insula (hyper-granular) and the IPL, as well as underlying white matter (Max_LSM_ [−34, −24, 0] IFOF). Simple sentence comprehension was associated with more inferior portions of the temporal lobe, namely the posterior and temporo-occipital divisions of the ITG and the MTG (Max_LSM_ [−62, −38, −6] MTG: caudal area 21). Conversely, the processing of more complex sentences involved the more superior portions of the temporal lobe such as the left STG, STS, the mid-posterior MTG and underlying white matter (Max_LSM_ passives: [−38, −24, −6], IFOF/ILF; subject extraction: [−34, −24, 0] IFOF and object extraction: [−46, −12, −8] ILF). In sum, the results of the extra analysis aligned with the previous analyses, further reinforcing our interpretation that the results reliably reflect syntactic deficits.

### Tract disconnection analyses

**Figure 5:**
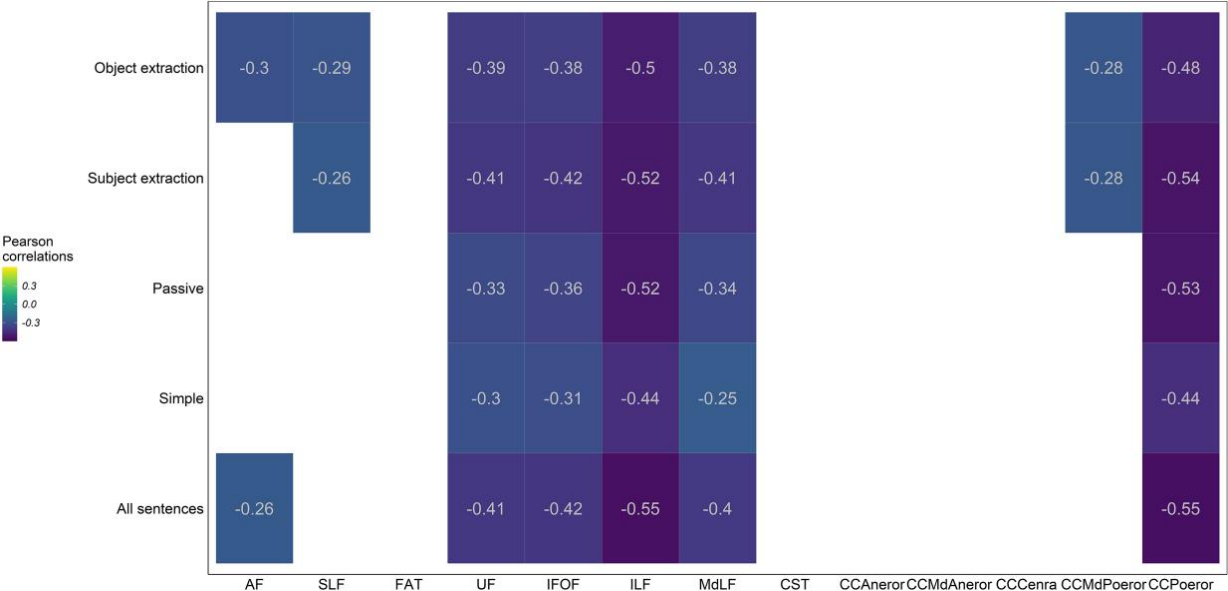
**White matter disconnection analysis.** Pearson correlations testing the impact of tract disconnection severity on the comprehension of different types of sentences. Negative values represent inverse correlations: the higher the disconnection of the tract, the lower the comprehension accuracy. The coloured cells indicate the correlations that survived the correction for multiple comparisons (Bonferroni-corrected alpha = 0.0038).

Correlation analyses between the comprehension accuracy of different types of sentences and the percentage of disconnection for different white matter tracts are reported in Fig. 5.

Impaired performance in overall sentence comprehension was associated with disconnection of the left ILF, IFOF, MdLF, UF and AF and with the disconnection of the more CCPoeor. However, when different sentence groups were considered separately, the correlation analyses showed a more nuanced picture. The disconnection of the left ILF, IFOF, MdLF and UF and the disconnection of the CCPoeor did appear to impact the comprehension of both canonical and non-canonical sentences. Interestingly, the disconnection of the left SLF and AF only affected the comprehension of certain types of complex sentence structures: the disconnection of the SLF impacted the comprehension of sentences with subject extraction, while the disconnection of both the SLF and the AF affected the comprehension of sentences containing object extraction. Comprehension of passive sentences was not significantly affected by the disconnection of any dorsal tracts. Similarly, the disconnection of the CCMdPoeor was detrimental for the comprehension of subject and object extractions, but not for passive sentences.

## Discussion

This study aimed to provide a comprehensive investigation of the neural basis of sentence comprehension, with a focus on understanding the roles of specific brain regions and white matter pathways in processing sentences with varying levels of syntactic complexity. To achieve these aims, we analysed auditory sentence comprehension data from a large cohort of left hemisphere stroke survivors and related performance to neural substrates using univariate and multivariate LSM as well as indirect structural disconnection mapping. In the following sections, we discuss the implications of our findings, first focusing on the distinct brain regions and white matter pathways that support general sentence-level comprehension and those that support comprehension of specific types of complex syntactic structures. We compare our results with previous LSM studies, highlighting both consistencies and unexpected findings. Next, we discuss the importance of considering a more nuanced definition of syntactic complexity, rather than relying simply on the canonical/non-canonical distinction. Finally, we discuss the implications of our findings for the formalization of reliable neurocognitive models of sentence comprehension.

### The role of temporal regions and underlying white matter pathways for successful sentence comprehension

One of the primary aims of our study was to identify the brain regions and white matter pathways that are critical for general sentence-level comprehension. All LSM analyses revealed consistent involvement of temporal regions for overall sentence comprehension performance, namely the left STS, mid-to-posterior MTG, STG and ITG. This finding aligns with previous empirical findings^11,17-28^ and with existing theoretical accounts^3-6^ that underline the crucial role of these left temporal regions during simple and complex sentence structure building: the posterior STG helps integrate phonological and syntactic information, the posterior ITG represents lexical-semantic information, while the posterior MTG helps integrate semantic and syntactic information and process more complex sentence structures, similarly to the posterior STS.

Building on previous LSM studies, which focused primarily on the role of grey matter regions in syntactic comprehension, a significant advancement in our study was analysing the role of different white matter tracts. Our findings showed that the disconnection of the left ILF, IFOF, MdLF, UF and AF impacted auditory comprehension of all sentence types, highlighting their critical roles in supporting general sentence-level comprehension.^3,4,36^ When different sentence types were analysed separately, the disconnection of the AF was specifically associated with impairments on the most complex sentence type only, object extraction. This result is further discussed in the next section. Finally, lesions in the CCPoeor also affected the comprehension of all sentence types. Prior studies have shown that, when integration demands increase, the right hemisphere^40^ and in particular posterior transcallosal white matter connections can play an important role, for example during word retrieval in sentences^32^ or during the integration of syntactic and prosodic information.^46^ Agenesis of the CC can also hinder the acquisition of syntactic comprehension skills during childhood.^45^ All these findings suggest that inter-hemispheric connections between the temporal lobes are also important for successful comprehension^32,40,45-47^ and that enriching current LSM analyses with indirect structural disconnection analyses may be beneficial in further characterizing the neural underpinnings of language comprehension.

### Frontal involvement and syntactic comprehension in LSM studies

There has been considerable debate in the literature about the involvement of left frontal regions in syntactic comprehension, particularly the pIFG or Broca’s area. We attributed the heterogeneity of previous findings to methodological differences, which in our study were addressed by investigating a large cohort of post-stroke individuals, employing both univariate and multivariate LSM analyses,^51^ considering white matter disconnection in correlation with behaviour,^54^ and, especially, by grouping sentences based on their different linguistic properties.

In the current study, the left inferior frontal regions, including Broca’s area, were not found to be critical for syntactic comprehension. This finding is in line with previous LSM studies that, like the current study, included critical covariates such as lesion size in the analyses.^17,21-23,25-27^ Previous studies that have reported a role for left inferior frontal cortex in syntactic comprehension did not correct for lesion size.^11,18,20^ To further investigate this discrepancy surrounding Broca’s area, we also ran univariate and multivariate LSM analyses without correcting for lesion volume (Supplementary Table 2 and Fig. 2). Here, portions of pars orbitalis (lateral area 12/47) of the left IFG emerge as relevant for the comprehension of both simple and complex sentences. Portions of Broca’s area, pars triangularis, of the left IFG (rostral area 45) did emerge as relevant for the comprehension of more complex sentences involving subject and object extractions (‘The girl is pulling the boy who is mad’, ‘It’s the boy that the girl is pulling’). Interestingly, there was very limited or no contribution of Broca’s area for the comprehension of simple declarative sentences or passives (e.g. ‘The girl is pulling the boy’, ‘The boy is being pulled by the girl’). This finding further suggests that, independently from methodological reasons (such as including lesion size as a covariate), the left pIFG might not be as crucial as expected for basic sentence structure building involving syntactic operations such as merge or unification, as predicted by some neurocognitive models of sentence comprehension.^3,4,8^

Although the left IFG was not critically related to syntactic comprehension in our study, our results showed that the disconnection of fibre tracts involving left frontal areas did significantly impact the comprehension of specific sentence types. The fact that only certain sentence types are affected by fronto-temporal white matter lesions also emphasizes the importance of considering a more nuanced definition of syntactic complexity^9^ when evaluating the contributions of different brain regions. These results are discussed in detail in the next section.

### Beyond the canonical versus non-canonical distinction: a finer-grained analysis of syntactic complexity

Our study addressed limitations in previous studies by categorizing various types of canonical and non-canonical sentences into more linguistically nuanced groupings, as a simple division between canonical and non-canonical sentences could be too coarse grained to fully capture different mechanisms and neural underpinnings necessary for successful syntactic comprehension. In this study, we considered different complex sentences: sentences containing passives (e.g. ‘The boy is pulled by the girl’), subject extraction (e.g. ‘The girl is pulling the boy who is mad’) and object extraction (e.g. ‘It’s the boy that the girl is pulling’). As we pointed out in the introduction, the successful comprehension of these sentence types relies on two core mechanisms, namely thematic role assignment and long-distance retrieval, and for this reason, the areas recruited for their processing might be different. In particular, among those structures, object extraction was expected to be the most demanding sentence type in terms of processing costs.^9,31^

Our LSM analyses and disconnection analyses highlighted the recruitment of different cortical regions in the temporal lobe and white matter connections for the successful comprehension of these different sentence types, even when accounting for lexical-semantic processing abilities. The comprehension of simple declarative sentences was associated with more inferior portions of the temporal lobe, namely the posterior portion of the ITG and the MTG as well as spared left temporal white matter tracts (ILF, IFOF, MdLF and UF). Interestingly, the disconnection of the SLF impacted comprehension of subject- and object-extracted sentences, but not passive sentences. These non-canonical sentence types are typically grouped together in LSM studies,^18-21,23,25-27^ thus obscuring the fact that they rely on different neural substrates. Successful thematic role assignment for the interpretation of passives can be achieved with spared left posterior temporal regions and underlying white matter tracts. Successful retrieval of the subject and object can be achieved if tracts that connect the left temporal and frontal regions are also spared. Finally, spared connections between the posterior STS and the pIFG via the AF were critical for comprehension of object-extracted sentences. This finding is in line with previous work suggesting that connections between the left temporal and frontal regions are critical for the processing of more complex sentence structures,^35,74^ arguably because of the recruitment of memory retrieval resources.^10,14,15,31^

Collectively, these findings underscore the significance of carefully considering and refining syntactic complexity for a better understanding of syntactic comprehension. By integrating LSM analyses with disconnection analyses and distinguishing various types of non-canonical sentences, we gain deeper insights into how the language network is engaged, depending on the specific processes at play.

### Implications for current neurocognitive models of sentence comprehension

Our findings have implications for refining neurocognitive models of language processing by providing empirical insights into the neural mechanisms underlying syntactic comprehension. Our findings support theoretical accounts highlighting the importance of left mid-to-posterior temporal cortex and underlying white matter tracts for syntactic structure building.^6,11,32,74,75^ Our findings are not consistent with prior theoretical accounts suggesting that the pIFG (Broca’s area) is the crucial hub for syntactic processing.^3,4,8,9^ Finally, our identification of distinct left cortical regions and white matter pathways involved in processing sentences with varying levels of syntactic complexity challenges the simplistic dichotomy between canonical and non-canonical sentence classifications. The involvement of more inferior left temporal regions in the processing of simple sentences and more superior temporal regions in the processing of more complex sentences and the involvement of different tracts connecting the frontal and temporal regions for the processing of very specific types of complex sentences suggest a gradient of syntactic complexity that should be considered in future research and current formalizations of sentence comprehension in the brain.

### Limitations and future directions

This study, while illuminating the neural correlates of syntactic comprehension, has a number of specific limitations. The current study utilized the CYCLE-R, which was developed to test comprehension on a wide range of syntactic structures but has a small number of test stimuli per subtest and a non-exhaustive set of sentence types. Also, although the CYCLE-R controls for a number of linguistic variables across sentence types (e.g. word frequency), other variables are not controlled (e.g. sentence length), thus limiting the ability of creating controlled minimal pairs.^9^ Another limitation is that our disconnection analyses relied on indirect measures of structural disconnection, as diffusion tensor imaging data were only available for a subset of participants. Diffusion tensor imaging data collection is now a part of our standard protocol, and we hope we can offer more reliable diffusion tensor imaging–based disconnection analyses in the future. Additionally, our dataset did not include information about the error types thus preventing us to provide error-based analyses and further characterize participants’ behaviour and type of processing deficits involved during the comprehension of different sentence structures. Moving away from aggregated scores, providing detailed by-item (error) information, expanding the concept of syntactic complexity to include a broader array of sentences and adopting even more mechanistic classifications such as quantifying memory retrieval difficulties for each type of sentence could offer richer insights. We believe that mechanism-based approaches can better address the so-called ‘granularity mismatch problem’,^76^ that is the challenge for reconciling nuanced, fine-grained linguistic operations and neuroscientific broader conceptual approaches to language.

Finally, the generalizability of these findings is subject to certain limitations related to the specific population studied. Future research should aim to test such finer-grained levels of syntactic complexity in more diverse populations. For instance, conducting similar studies in non-English speaking populations would help determine if our findings are consistent across languages with different syntactic structures and linguistic features.

In sum, while this study contributes valuable insights into the neural underpinnings of syntactic comprehension, it also opens several avenues for future research. By addressing these limitations and pursuing the outlined directions, subsequent studies can further elucidate the intricate relationship between brain function and syntactic processing, enhancing our understanding of the neural architecture of language.

## Conclusion

This comprehensive study explored the neural foundations of syntactic comprehension, particularly investigating the roles of specific brain regions and white matter pathways in processing sentences with varying syntactic complexity. Our findings spotlight the critical role of left posterior temporal regions and associated white matter pathways in syntactic comprehension, challenging traditional views on the predominant role of the left inferior frontal regions in this domain. Through a nuanced examination of syntactic complexity, we have achieved a more refined understanding of the neural architecture underlying sentence comprehension. These findings offer novel insights for future research and refining current neurocognitive models of sentence comprehension.

## Supplementary Material

fcae379_Supplementary_Data

## Data Availability

Raw data were generated at the VA Northern California Health Care System and at the UC Berkeley. Results supporting the findings of this study and the codes generated are available in Open Science Framework (OSF: https://osf.io/7zekw/).
