# Supplementary material for: Mapping sentence comprehension and syntactic complexity: evidence from 131 stroke survivors
Source: Brain Commun. 2024 Nov 15;6(6):fcae379. doi: 10.1093/braincomms/fcae379 (PMC11565230; doi:10.1093/braincomms/fcae379)
Supplement: fcae379_Supplementary_Data [file fcae379_supplementary_data.pdf]

Supplementary Materials

Supplementary Table 1. Parcels identified in the LSM analysis.

Brainnetome Atlas parcels identified by the univariate LSM analyses and multivariate LSM analyses with lesion size as a covariate. When no parcels had overlap above 10%, we report all parcels covered by the LSM cluster irrespective of their coverage.

Sentence group	*Analysis*	*BNA Parcel Name*	*Acronym*	*% Overlap*
All sentence types	*Univariate*	rpSTS, rostroposterior superior temporal sulcus	pSTS	91
		cpSTS, caudoposterior superior temporal sulcus	pSTS	71
		A22r, rostral area 22	STG	66
		A37dl, dorsolateral area37	MTG	64
		aSTS, anterior superior temporal sulcus	aSTS	63
		A41/42, area 41/42	STG	55
		A21c, caudal area 21	MTG	49
		A22c, caudal area 22	STG	48
		A39rv, rostroventral area 39(PGa)	IPL	23
		TE1.0 and TE1.2	STG	17
		G, hypergranular insula	INS	13
		A37vl, ventrolateral area 37	ITG	13
		A20cl, caudolateral of area 20	ITG	12
		A39c, caudal area 39(PGp)	IPL	11
	Multivariate	A22r, rostral area 22	STG	5
		A41/42, area 41/42	STG	5
		aSTS, anterior superior temporal sulcus	aSTS	4
		G, hypergranular insula	INS	2
		rpSTS, rostroposterior superior temporal sulcus	pSTS	1
Simple	*Univariate*	A21c, caudal area 21	MTG	84
		A37vl, ventrolateral area 37	ITG	77
		A20cl, caudolateral of area 20	ITG	73
		A37dl, dorsolateral area37	MTG	67
		V5/MT+, area V5/MT+	LOcC	56
		rpSTS, rostroposterior superior temporal sulcus	pSTS	46
		aSTS, anterior superior temporal sulcus	aSTS	38
		cpSTS, caudoposterior superior temporal sulcus	pSTS	32
		A39c, caudal area 39(PGp)	IPL	18

		A37elv, extreme lateroventral area37	ITG	16
		A22c, caudal area 22	STG	10
	Multivariate	A21c, caudal area 21	MTG	66
		A20cl, caudolateral of area 20	ITG	38
		A37vl, ventrolateral area 37	ITG	24
		aSTS, anterior superior temporal sulcus	aSTS	13
		A37dl, dorsolateral area37	MTG	10
Passive	*Univariate*	rpSTS, rostroposterior superior temporal sulcus	pSTS	96
		aSTS, anterior superior temporal sulcus	aSTS	76
		A22r, rostral area 22	STG	64
		A21c, caudal area 21	MTG	62
		A37dl, dorsolateral area37	MTG	59
		cpSTS, caudoposterior superior temporal sulcus	pSTS	56
		A39c, caudal area 39(PGp)	IPL	44
		A41/42, area 41/42	STG	36
		A22c, caudal area 22	STG	35
		A37vl, ventrolateral area 37	ITG	28
		A20cl, caudolateral of area 20	ITG	28
		V5/MT+, area V5/MT+	LOcC	19
		A39rv, rostroventral area 39(PGa)	IPL	14
		TE1.0 and TE1.2	STG	12
		G, hypergranular insula	INS	12
	Multivariate	aSTS, anterior superior temporal sulcus	aSTS	4
		G, hypergranular insula	INS	1
		A22r, rostral area 22	STG	1
Subject extraction	*Univariate*	rpSTS, rostroposterior superior temporal sulcus	pSTS	76
		cpSTS, caudoposterior superior temporal sulcus	pSTS	49
		A41/42, area 41/42	STG	38
		A22r, rostral area 22	STG	38
		aSTS, anterior superior temporal sulcus	aSTS	28
		A37dl, dorsolateral area37	MTG	24
		A22c, caudal area 22	STG	21
		TE1.0 and TE1.2	STG	11
	Multivariate	A41/42, area 41/42	STG	2
		A22r, rostral area 22	STG	2
		aSTS, anterior superior temporal sulcus	aSTS	1
		rpSTS, rostroposterior superior temporal sulcus	pSTS	1
Object extraction	*Univariate*	aSTS, anterior superior temporal sulcus	aSTS	26
		A21c, caudal area 21	MTG	19
		rpSTS, rostroposterior superior temporal sulcus	pSTS	14

Multivariate analyses

Multivariate SVR-LSM analyses identified significant LSM clusters in 4 out of the 5 sentence group analyses (see Figure A1). Overall, all sentence groups were associated with the superior portion of the temporal lobe, namely the STG and the STS, as well as underlying white matter (Max_{LSM} [-40, -24, -6] ILF/IFOF). In the analyses considering different levels of syntactic complexity separately, simple sentence comprehension was associated with the mid-posterior portion of the MTG and the posterior portion of the ITG (Max_{LSM} Simple: [-70, -20, -16] MTG: caudal area 21), while the more anterior portion of the STG and STS were noted as crucial for the processing of more complex sentences (Passive: [-38, -22, -6] IFOF/ILF; Subject extraction: [-40, -26, -0] STG: TE1.0 and TE1.2).

Supplementary Figure 1. Multivariate LSM analysis.

SVR LSM maps for the comprehension of different groups of sentences from the CYCLE-R test, with age, months post-onset, education, and lesion volume as covariates. The colored bar represents the range of significant SVR beta-values following permutation-based correction for multiple comparisons.

Multivariate LSM
(covariates: age, education, time post-stroke, lesion size)

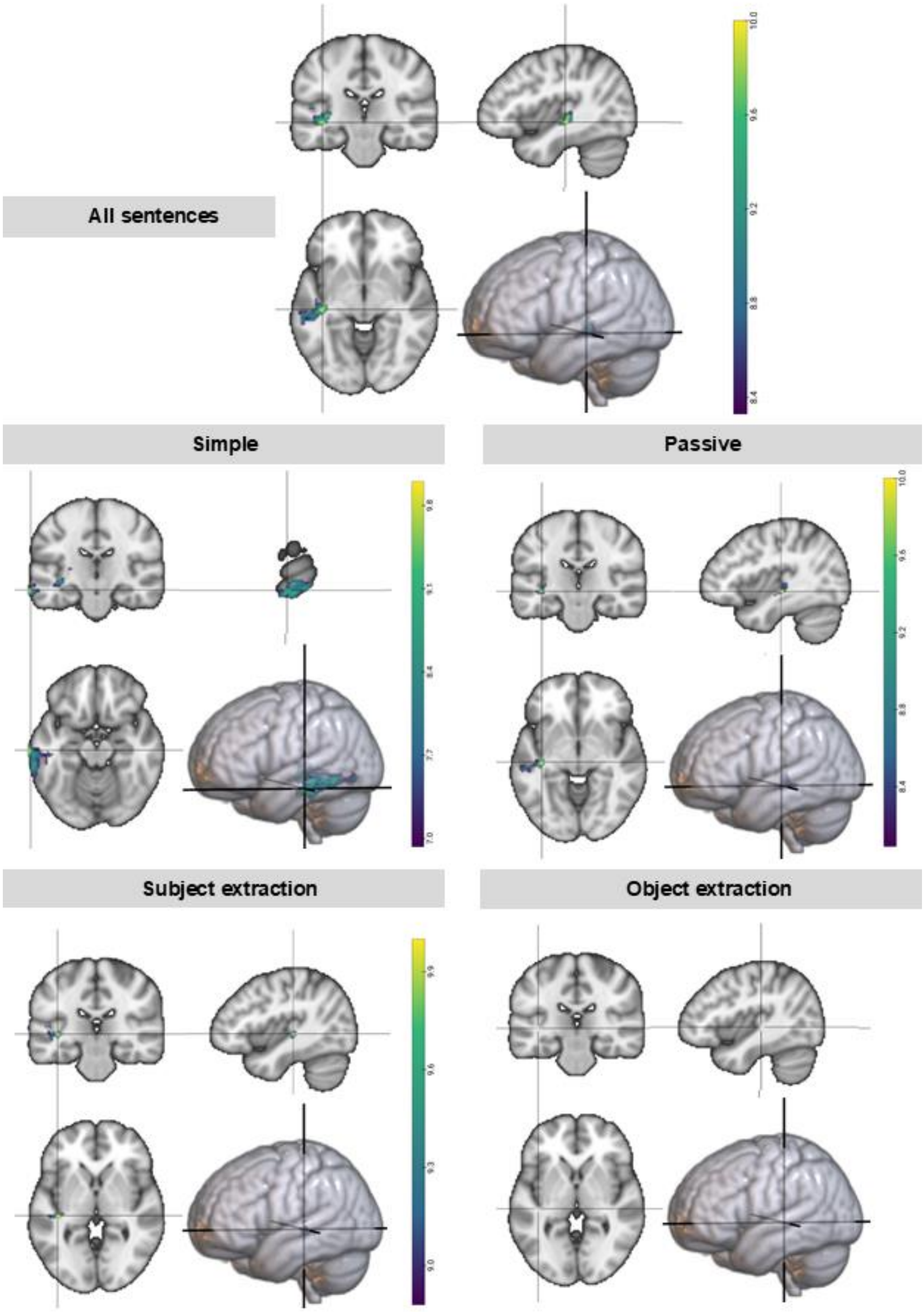

Supplementary Table 2. Parcels identified in the LSM analyses without lesion volume correction.

Brainnetome Atlas parcels identified by the univariate LSM analyses and multivariate LSM analyses without lesion size as a covariate. We also report the maximum statistic location (MaxLSM), namely the peak coordinates ([px, py, pz]) of the significant cluster identified by each analysis. When no parcels had overlap above 10%, we report all parcels covered by the LSM cluster irrespective of their coverage.

Sentence group	*Analysis*	*BNA Parcel Name*	*Acronym*	*% Overlap*
All sentence types	*Univariate* [-38, -22, -6] IFOF/ILF	A41/42, area 41/42	STG	100
		rpSTS, rostroposterior superior temporal sulcus	pSTS	100
		cpSTS, caudoposterior superior temporal sulcus	pSTS	99
		vId/vIg, ventral dysgranular and granular insula	INS	99
		A37vl, ventrolateral area 37	ITG	98
		A22r, rostral area 22	STG	97
		A39rv, rostroventral area 39(PGa)	IPL	96
		aSTS, anterior superior temporal sulcus	aSTS	94
		TE1.0 and TE1.2	STG	94
		A20cl, caudolateral of area 20	ITG	93
		A37dl, dorsolateral area37	MTG	91
		G, hypergranular insula	INS	90
		V5/MT+, area V5/MT+	LOcC	90
		vIa, ventral agranular insula	INS	89
		A21c, caudal area 21	MTG	88
		A40rv, rostroventral area 40(PFop)	IPL	82
		A39c, caudal area 39(PGp)	IPL	81
		A12/47l, lateral area 12/47	OrG	81
		A39rd, rostrodorsal area 39(Hip3)	IPL	80
		A22c, caudal area 22	STG	79
		A40rd, rostrodorsal area 40(PFt)	IPL	75
		A40c, caudal area 40(PFm)	IPL	65
		A38l, lateral area 38	STG	64
		A12/47o, orbital area 12/47	OrG	63
		A45r, rostral area 45	IFG	61
		lAmyg, lateral amygdala	Amyg	52
		A20cv, caudoventral of area 20	ITG	48
		A37elv, extreme lateroventral area37	ITG	46
		GP, globus pallidus	BG	41
		A5l, lateral area 5	SPL	39

	A7ip, intraparietal area 7(hIP3)	SPL	36
	A2, area 2	PoG	31
	IFS, inferior frontal sulcus	IFG	28
	NAC, nucleus accumbens	BG	27
	Stha, sensory thalamus	Tha	27
	dlPu, dorsolateral putamen	BG	26
	A44d,dorsal area 44	IFG	26
	A11l, lateral area 11	OrG	24
	dIg, dorsal granular insula	INS	23
	cHipp, caudal hippocampus	Hipp	22
	vmPu, ventromedial putamen	BG	21
	A20il, intermediate lateral area 20	ITG	21
	mPMtha, pre-motor thalamus	Tha	20
	A1/2/3ulhf, area 1/2/3(upper limb, head and face region)	PoG	20
	A4ul, area 4(upper limb region)	PrG	18
	A37lv, lateroventral area37	FuG	16
	A1/2/3tonIa, area 1/2/3(tongue and larynx region)	PoG	16
	vCa, ventral caudate	BG	16
	A20iv, intermediate ventral area 20	ITG	15
	IFJ, inferior frontal junction	MFG	15
	A21r, rostral area 21	MTG	13
	dId, dorsal dysgranular insula	INS	11
	lPFtha, lateral pre-frontal thalamus	Tha	11
	mOccG, middle occipital gyrus	LOcC	10
	mAmyg, medial amygdala	Amyg	10
	dCa, dorsal caudate	BG	10
	A9/46v, ventral area 9/46	MFG	10
Multivariate	A41/42, area 41/42	STG	100
[-36, -24, -2] Hypergranular insula	rpSTS, rostroposterior superior temporal sulcus	pSTS	100
	cpSTS, caudoposterior superior temporal sulcus	pSTS	99
	vId/vIg, ventral dysgranular and granular insula	INS	98
	A39rv, rostroventral area 39(PGa)	IPL	96
	A22r, rostral area 22	STG	96
	aSTS, anterior superior temporal sulcus	aSTS	94
	A37dl, dorsolateral area37	MTG	91
	G, hypergranular insula	INS	90
	TE1.0 and TE1.2	STG	88
	A21c, caudal area 21	MTG	86
	A40rv, rostroventral area 40(PFop)	IPL	77
	A37vl, ventrolateral area 37	ITG	76
	A22c, caudal area 22	STG	75

		A39c, caudal area 39(PGp)	IPL	69
		A40rd, rostrodorsal area 40(PFt)	IPL	67
		A40c, caudal area 40(PFm)	IPL	66
		A38l, lateral area 38	STG	65
		A39rd, rostrodorsal area 39(Hip3)	IPL	64
		A20cl, caudolateral of area 20	ITG	63
		vIa, ventral agranular insula	INS	52
		lAmyg, lateral amygdala	Amyg	46
		V5/MT+, area V5/MT+	LOcC	44
		A12/47l, lateral area 12/47	OrG	40
		A5l, lateral area 5	SPL	25
		A12/47o, orbital area 12/47	OrG	22
		NAC, nucleus accumbens	BG	22
		A45r, rostral area 45	IFG	22
		A2, area 2	PoG	20
		A20il, intermediate lateral area 20	ITG	19
		GP, globus pallidus	BG	19
		dlPu, dorsolateral putamen	BG	19
		dIg, dorsal granular insula	INS	18
		A11l, lateral area 11	OrG	16
		cHipp, caudal hippocampus	Hipp	14
		A1/2/3tonIa, area 1/2/3(tongue and larynx region)	PoG	14
		vmPu, ventromedial putamen	BG	12
		A7ip, intraparietal area 7(hIP3)	SPL	11
		A21r, rostral area 21	MTG	11
		dCa, dorsal caudate	BG	11
		A37elv, extreme lateroventral area 37	ITG	11
Simple	*Univariate*	rpSTS, rostroposterior superior temporal sulcus	pSTS	100
	[-70, -26, -8] MTG: caudal area 21	A37vl, ventrolateral area 37	ITG	98
		cpSTS, caudoposterior superior temporal sulcus	pSTS	98
		A20cl, caudolateral of area 20	ITG	97
		A22r, rostral area 22	STG	96
		A21c, caudal area 21	MTG	95
		aSTS, anterior superior temporal sulcus	aSTS	94
		A37dl, dorsolateral area37	MTG	90
		V5/MT+, area V5/MT+	LOcC	86
		A39rv, rostroventral area 39(PGa)	IPL	85
		A41/42, area 41/42	STG	77
		A39c, caudal area 39(PGp)	IPL	75
		A22c, caudal area 22	STG	63
		TE1.0 and TE1.2	STG	51

	A37elv, extreme lateroventral area37	ITG	50
	A38l, lateral area 38	STG	38
	G, hypergranular insula	INS	34
	vId/vIg, ventral dysgranular and granular insula	INS	33
	A20cv, caudoventral of area 20	ITG	33
	A39rd, rostrodorsal area 39(Hip3)	IPL	32
	A20il, intermediate lateral area 20	ITG	27
	A40c, caudal area 40(PFm)	IPL	21
	A12/47l, lateral area 12/47	OrG	21
	cHipp, caudal hippocampus	Hipp	15
	mOccG, middle occipital gyrus	LOcC	12
	A45r, rostral area 45	IFG	11
	A40rv, rostroventral area 40(PFop)	IPL	10
	A20iv, intermediate ventral area 20	ITG	10
Multivariate	rpSTS, rostroposterior superior temporal sulcus	pSTS	100
[-38, -22, -6] IFOF/ILF	cpSTS, caudoposterior superior temporal sulcus	pSTS	99
	A22r, rostral area 22	STG	95
	A41/42, area 41/42	STG	93
	aSTS, anterior superior temporal sulcus	aSTS	93
	A39rv, rostroventral area 39(PGa)	IPL	93
	A37vl, ventrolateral area 37	ITG	91
	A20cl, caudolateral of area 20	ITG	88
	A21c, caudal area 21	MTG	86
	A37dl, dorsolateral area37	MTG	86
	A39c, caudal area 39(PGp)	IPL	68
	A22c, caudal area 22	STG	67
	V5/MT+, area V5/MT+	LOcC	64
	TE1.0 and TE1.2	STG	58
	A39rd, rostrodorsal area 39(Hip3)	IPL	58
	G, hypergranular insula	INS	48
	A38l, lateral area 38	STG	43
	vId/vIg, ventral dysgranular and granular insula	INS	38
	A40c, caudal area 40(PFm)	IPL	37
	A40rv, rostroventral area 40(PFop)	IPL	30
	Stha, sensory thalamus	Tha	25
	A12/47l, lateral area 12/47	OrG	22
	lAmyg, lateral amygdala	Amyg	22
	A37elv, extreme lateroventral area37	ITG	21
	A20cv, caudoventral of area 20	ITG	18
	A20il, intermediate lateral area 20	ITG	18
	A45r, rostral area 45	IFG	17
	cHipp, caudal hippocampus	Hipp	14
	GP, globus pallidus	BG	14

		NAC, nucleus accumbens	BG	11
Passive	*Univariate:* [-38, -24, -6] IFOF/ILF	rpSTS, rostroposterior superior temporal sulcus	pSTS	100
		cpSTS, caudoposterior superior temporal sulcus	pSTS	99
		A41/42, area 41/42	STG	98
		A39rv, rostroventral area 39(PGa)	IPL	98
		A22r, rostral area 22	STG	98
		A37vl, ventrolateral area 37	ITG	97
		aSTS, anterior superior temporal sulcus	aSTS	96
		A21c, caudal area 21	MTG	95
		A37dl, dorsolateral area37	MTG	93
		V5/MT+, area V5/MT+	LOcC	91
		A20cl, caudolateral of area 20	ITG	88
		A22c, caudal area 22	STG	82
		A39c, caudal area 39(PGp)	IPL	80
		TE1.0 and TE1.2	STG	75
		G, hypergranular insula	INS	68
		A39rd, rostrodorsal area 39(Hip3)	IPL	67
		vId/vIg, ventral dysgranular and granular insula	INS	55
		A38l, lateral area 38	STG	52
		A40c, caudal area 40(PFm)	IPL	51
		A37elv, extreme lateroventral area37	ITG	43
		A40rv, rostroventral area 40(PFop)	IPL	42
		lAmyg, lateral amygdala	Amyg	40
		A20cv, caudoventral of area 20	ITG	39
		A37lv, lateroventral area37	FuG	20
		cHipp, caudal hippocampus	Hipp	19
		A20il, intermediate lateral area 20	ITG	19
		A20iv, intermediate ventral area 20	ITG	16
		A12/47l, lateral area 12/47	OrG	15
		A7ip, intraparietal area 7(hIP3)	SPL	14
		GP, globus pallidus	BG	13
		Stha, sensory thalamus	Tha	13
		A5l, lateral area 5	SPL	12
		mOccG, middle occipital gyrus	LOcC	11
		NAC, nucleus accumbens	BG	11
		A21r, rostral area 21	MTG	11
		A11l, lateral area 11	OrG	10
	Multivariate	A41/42, area 41/42	STG	100
	[-38, -22, -6] IFOF/ILF	rpSTS, rostroposterior superior temporal sulcus	pSTS	100
		cpSTS, caudoposterior superior temporal sulcus	pSTS	99
		A22r, rostral area 22	STG	96

		A39rv, rostroventral area 39(PGa)	IPL	95
		aSTS, anterior superior temporal sulcus	aSTS	94
		A37dl, dorsolateral area 37	MTG	88
		A21c, caudal area 21	MTG	85
		TE1.0 and TE1.2	STG	82
		G, hypergranular insula	INS	77
		A22c, caudal area 22	STG	74
		A39c, caudal area 39(PGp)	IPL	68
		vId/vIg, ventral dysgranular and granular insula	INS	64
		A20cl, caudolateral of area 20	ITG	57
		A38l, lateral area 38	STG	57
		A37vl, ventrolateral area 37	ITG	55
		A40rv, rostroventral area 40(PFop)	IPL	50
		A40c, caudal area 40(PFm)	IPL	49
		A39rd, rostrodorsal area 39(Hip3)	IPL	49
		lAmyg, lateral amygdala	Amyg	36
		V5/MT+, area V5/MT+	LOcC	25
		A20il, intermediate lateral area 20	ITG	19
		A5l, lateral area 5	SPL	14
		A40rd, rostrodorsal area 40(PFt)	IPL	13
		vIa, ventral agranular insula	INS	12
		A21r, rostral area 21	MTG	11
		NAC, nucleus accumbens	BG	10
		cHipp, caudal hippocampus	Hipp	10
Subject extraction	*Univariate* [-34, -24, 0] IFOF	A41/42, area 41/42	STG	100
		rpSTS, rostroposterior superior temporal sulcus	pSTS	100
		cpSTS, caudoposterior superior temporal sulcus	pSTS	99
		A39rv, rostroventral area 39(PGa)	IPL	98
		vId/vIg, ventral dysgranular and granular insula	INS	98
		A22r, rostral area 22	STG	98
		aSTS, anterior superior temporal sulcus	aSTS	96
		A21c, caudal area 21	MTG	93
		A37dl, dorsolateral area37	MTG	92
		G, hypergranular insula	INS	88
		A37vl, ventrolateral area 37	ITG	84
		TE1.0 and TE1.2	STG	82
		A22c, caudal area 22	STG	81
		A20cl, caudolateral of area 20	ITG	79
		A40rv, rostroventral area 40(PFop)	IPL	72
		A38l, lateral area 38	STG	67
		A39c, caudal area 39(PGp)	IPL	64
		A40c, caudal area 40(PFm)	IPL	63

	vIa, ventral agranular insula	INS	61
	lAmyg, lateral amygdala	Amyg	60
	A12/47l, lateral area 12/47	OrG	58
	A40rd, rostrodorsal area 40(PFt)	IPL	53
	A39rd, rostrodorsal area 39(Hip3)	IPL	50
	A45r, rostral area 45	IFG	48
	A12/47o, orbital area 12/47	OrG	36
	V5/MT+, area V5/MT+	LOcC	34
	NAC, nucleus accumbens	BG	32
	GP, globus pallidus	BG	27
	vmPu, ventromedial putamen	BG	25
	cHipp, caudal hippocampus	Hipp	23
	A5l, lateral area 5	SPL	22
	dlPu, dorsolateral putamen	BG	21
	A37elv, extreme lateroventral area37	ITG	20
	A20il, intermediate lateral area 20	ITG	20
	dIg, dorsal granular insula	INS	19
	mAmyg, medial amygdala	Amyg	18
	vCa, ventral caudate	BG	18
	A1/2/3tonIa, area 1/2/3(tongue and larynx region)	PoG	18
	A11l, lateral area 11	OrG	18
	A2, area 2	PoG	17
	A20cv, caudoventral of area 20	ITG	15
	dCa, dorsal caudate	BG	13
	A21r, rostral area 21	MTG	11
	Stha, sensory thalamus	Tha	11
	A20iv, intermediate ventral area 20	ITG	10
Multivariate	A41/42, area 41/42	STG	100
[-40, -26, 0] STG: TE1.0 and TE1.2	rpSTS, rostroposterior superior temporal sulcus	pSTS	100
	cpSTS, caudoposterior superior temporal sulcus	pSTS	99
	A22r, rostral area 22	STG	96
	vId/vIg, ventral dysgranular and granular insula	INS	96
	A39rv, rostroventral area 39(PGa)	IPL	96
	aSTS, anterior superior temporal sulcus	aSTS	94
	A37dl, dorsolateral area37	MTG	89
	G, hypergranular insula	INS	85
	TE1.0 and TE1.2	STG	84
	A21c, caudal area 21	MTG	83
	A40rv, rostroventral area 40(PFop)	IPL	73
	A22c, caudal area 22	STG	73
	A38l, lateral area 38	STG	62
	A39c, caudal area 39(PGp)	IPL	61
	A37vl, ventrolateral area 37	ITG	58

		A40c, caudal area 40(PFm)	IPL	58
		A40rd, rostrodorsal area 40(PFt)	IPL	56
		vIa, ventral agranular insula	INS	55
		A20cl, caudolateral of area 20	ITG	49
		A12/47l, lateral area 12/47	OrG	49
		lAmyg, lateral amygdala	Amyg	44
		A45r, rostral area 45	IFG	32
		A39rd, rostrodorsal area 39(Hip3)	IPL	32
		A12/47o, orbital area 12/47	OrG	29
		NAC, nucleus accumbens	BG	27
		vmPu, ventromedial putamen	BG	23
		A5l, lateral area 5	SPL	20
		A20il, intermediate lateral area 20	ITG	18
		V5/MT+, area V5/MT+	LOcC	17
		A11l, lateral area 11	OrG	17
		A2, area 2	PoG	16
		dlPu, dorsolateral putamen	BG	16
		cHipp, caudal hippocampus	Hipp	15
		dIg, dorsal granular insula	INS	15
		vCa, ventral caudate	BG	14
		A1/2/3tonIa, area 1/2/3(tongue and larynx region)	PoG	14
		A21r, rostral area 21	MTG	10
Object extraction	*Univariate* [-60, -22, -8] aSTS	A41/42, area 41/42	STG	100
		rpSTS, rostroposterior superior temporal sulcus	pSTS	100
		vId/vIg, ventral dysgranular and granular insula	INS	100
		cpSTS, caudoposterior superior temporal sulcus	pSTS	99
		A37vl, ventrolateral area 37	ITG	98
		A22r, rostral area 22	STG	96
		aSTS, anterior superior temporal sulcus	aSTS	94
		A39rv, rostroventral area 39(PGa)	IPL	93
		G, hypergranular insula	INS	93
		TE1.0 and TE1.2	STG	87
		A21c, caudal area 21	MTG	86
		A20cl, caudolateral of area 20	ITG	86
		A37dl, dorsolateral area37	MTG	85
		A40rv, rostroventral area 40(PFop)	IPL	85
		A40rd, rostrodorsal area 40(PFt)	IPL	85
		vmPu, ventromedial putamen	BG	82
		V5/MT+, area V5/MT+	LOcC	81
		GP, globus pallidus	BG	74
		A22c, caudal area 22	STG	72
		A39rd, rostrodorsal area 39(Hip3)	IPL	71

	A38l, lateral area 38	STG	67
	A40c, caudal area 40(PFm)	IPL	66
	vIa, ventral agranular insula	INS	65
	A39c, caudal area 39(PGp)	IPL	64
	A12/47l, lateral area 12/47	OrG	56
	dlPu, dorsolateral putamen	BG	56
	A5l, lateral area 5	SPL	54
	A12/47o, orbital area 12/47	OrG	54
	lAmyg, lateral amygdala	Amyg	52
	A7ip, intraparietal area 7(hIP3)	SPL	52
	A2, area 2	PoG	49
	A37elv, extreme lateroventral area37	ITG	43
	dCa, dorsal caudate	BG	39
	Stha, sensory thalamus	Tha	33
	A4ul, area 4(upper limb region)	PrG	30
	A1/2/3ulhf, area 1/2/3(upper limb, head and face region)	PoG	30
	A45r, rostral area 45	IFG	30
	NAC, nucleus accumbens	BG	28
	vCa, ventral caudate	BG	28
	dIg, dorsal granular insula	INS	27
	A20cv, caudoventral of area 20	ITG	24
	mPMtha, pre-motor thalamus	Tha	23
	A11l, lateral area 11	OrG	23
	A20il, intermediate lateral area 20	ITG	19
	A1/2/3tonIa, area 1/2/3(tongue and larynx region)	PoG	18
	lPFtha, lateral pre-frontal thalamus	Tha	17
	cHipp, caudal hippocampus	Hipp	14
	A21r, rostral area 21	MTG	12
	A6cdl, caudal dorsolateral area 6	PrG	12
	A37lv, lateroventral area37	FuG	12
	A6cvl, caudal ventrolateral area 6	PrG	12
	PPtha, posterior parietal thalamus	Tha	11
Multivariate	rpSTS, rostroposterior superior temporal sulcus	pSTS	100
[-46, -12, -8] ILF	A41/42, area 41/42	STG	99
	cpSTS, caudoposterior superior temporal sulcus	pSTS	98
	A22r, rostral area 22	STG	96
	aSTS, anterior superior temporal sulcus	aSTS	93
	vId/vIg, ventral dysgranular and granular insula	INS	93
	A39rv, rostroventral area 39(PGa)	IPL	92
	G, hypergranular insula	INS	88
	A21c, caudal area 21	MTG	82
	TE1.0 and TE1.2	STG	81

A40rd, rostrodorsal area 40(PFt)	IPL	73
A22c, caudal area 22	STG	69
A37dl, dorsolateral area37	MTG	67
A40rv, rostroventral area 40(PFop)	IPL	66
A38l, lateral area 38	STG	65
A40c, caudal area 40(PFm)	IPL	62
A37vl, ventrolateral area 37	ITG	52
A39rd, rostrodorsal area 39(Hip3)	IPL	49
A20cl, caudolateral of area 20	ITG	49
A5l, lateral area 5	SPL	46
A39c, caudal area 39(PGp)	IPL	41
lAmyg, lateral amygdala	Amyg	38
A7ip, intraparietal area 7(hIP3)	SPL	30
A2, area 2	PoG	28
dlPu, dorsolateral putamen	BG	28
vIa, ventral agranular insula	INS	26
NAC, nucleus accumbens	BG	20
GP, globus pallidus	BG	20
vmPu, ventromedial putamen	BG	20
dCa, dorsal caudate	BG	18
A20il, intermediate lateral area 20	ITG	16
dIg, dorsal granular insula	INS	12
V5/MT+, area V5/MT+	LOcC	12
A11l, lateral area 11	OrG	12
A1/2/3ulhf, area 1/2/3(upper limb, head and face region)	PoG	11
A21r, rostral area 21	MTG	10

Supplementary Figure 2. LSM analyses without lesion volume correction.

Univariate and SVR LSM maps for the comprehension of different groups of sentences from the CYCLE-R test, with age, months post-onset and education as covariates.

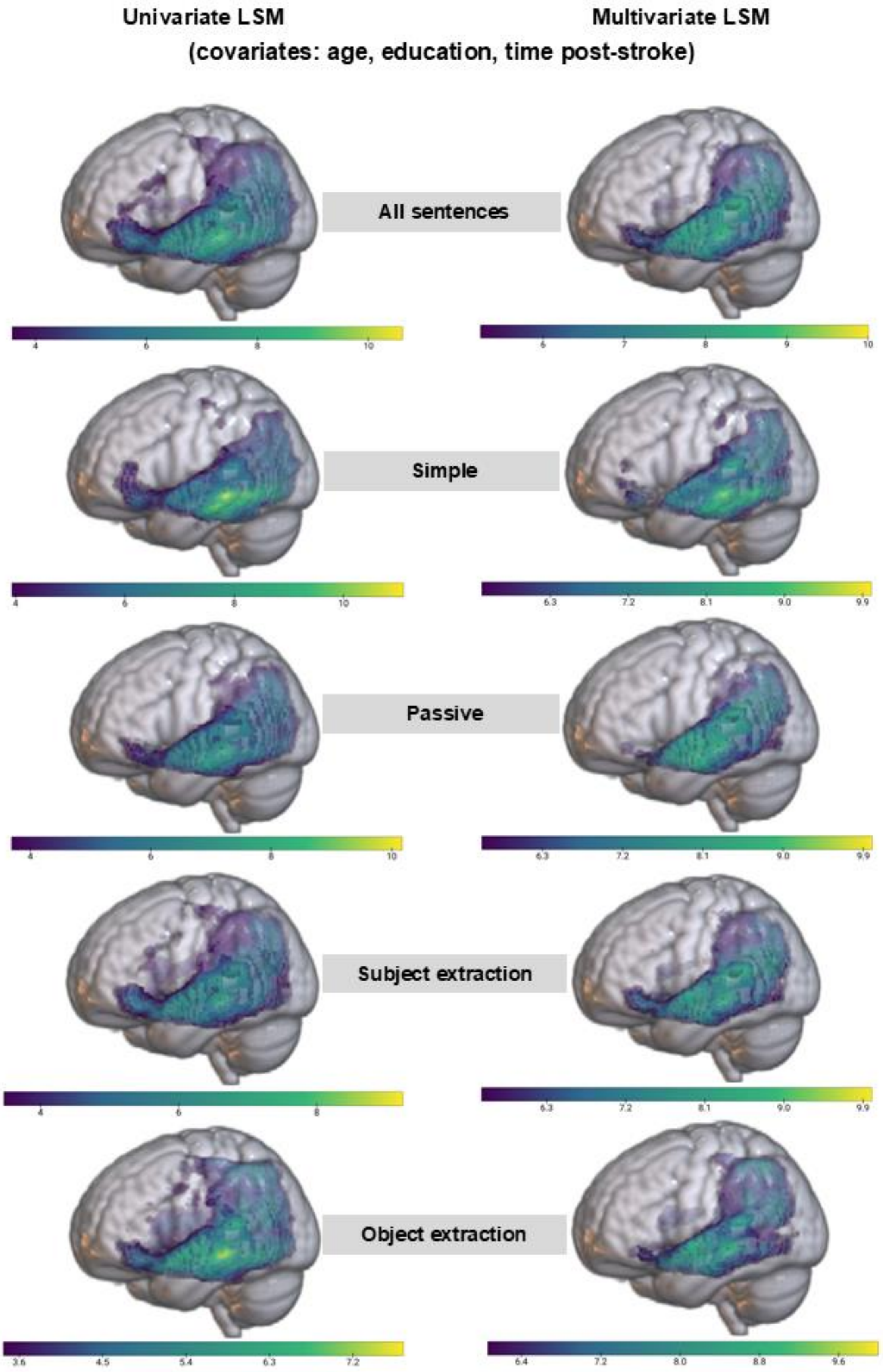

Univariate LSM
Multivariate LSM
(covariates: age, education, time post-stroke)
All sentences
4 6 8 10
6 7 8 9 10
Simple
4 6 8 10
6.3 7.2 8.1 9.0 9.9
Passive
4 6 8 10
6.3 7.2 8.1 9.0 9.9
Subject extraction
4 6 8
6.3 7.2 8.1 9.0 9.9
Object extraction
3.6 4.5 5.4 6.3 7.2
6.4 7.2 8.0 8.8 9.6

Supplementary Table 3. Parcels identified in analyses controlling for lexical-semantic processing.

Brainnetome Atlas parcels identified in the univariate LSM analysis including lexical-semantic processing as an additional covariate, together with age, education, time post-stroke, and lesion volume.

Sentence group	*BNA Parcel Name*	*Acronym*	*% Overlap*
All sentence types	A41/42, area 41/42	STG	53.1
	rpSTS, rostroposterior superior temporal sulcus	pSTS	48.3
	aSTS, anterior superior temporal sulcus	aSTS	37
	A22c, caudal area 22	STG	36.6
	TE1.0 and TE1.2	STG	31.2
	cpSTS, caudoposterior superior temporal sulcus	pSTS	20.1
	A21c, caudal area 21	MTG	17.6
	G, hypergranular insula	INS	17.1
	A39rv, rostroventral area 39(PGa)	IPL	15.8
	A22r, rostral area 22	STG	13
Simple	A21c, caudal area 21	MTG	47.9
	A20cl, caudolateral of area 20	ITG	45.1
	A37vl, ventrolateral area 37	ITG	38.6
	aSTS, anterior superior temporal sulcus	aSTS	10.8
Passive	A41/42, area 41/42	STG	38.6
	rpSTS, rostroposterior superior temporal sulcus	pSTS	26
	aSTS, anterior superior temporal sulcus	aSTS	25.3
	TE1.0 and TE1.2	STG	19.6
	A22c, caudal area 22	STG	17.9
	G, hypergranular insula	INS	16.5
	A22r, rostral area 22	STG	12.7
Subject extraction	A41/42, area 41/42	STG	47.5
	TE1.0 and TE1.2	STG	26.8
	A22c, caudal area 22	STG	25.1
	aSTS, anterior superior temporal sulcus	aSTS	19.3
	rpSTS, rostroposterior superior temporal sulcus	pSTS	19.1
	G, hypergranular insula	INS	11.6
Object extraction	aSTS, anterior superior temporal sulcus	aSTS	12.8
